# Biapenem as a Novel Insight into Drug Repositioning against Particulate Matter-Induced Lung Injury

**DOI:** 10.3390/ijms21041462

**Published:** 2020-02-21

**Authors:** Wonhwa Lee, Moon-Chang Baek, Kyung-Min Kim, Jong-Sup Bae

**Affiliations:** 1 College of Pharmacy, CMRI, Research Institute of Pharmaceutical Sciences, BK21 Plus KNU Multi-Omics based Creative Drug Research Team, Kyungpook National University, Daegu 41566, Korea; bywonhwalee@gmail.com; 2 Department of Molecular Medicine, CMRI, School of Medicine, Kyungpook National University, Daegu 41566, Korea; mcbaek@knu.ac.kr; 3 Division of Plant Biosciences, School of Applied BioSciences, College of Agriculture and Life Science, Kyungpook National University, Daegu 41566, Korea; kkm@knu.ac.kr

**Keywords:** drug repositioning, biapenem, particulate matter, lung injury, TLR4-mTOR-autophagy

## Abstract

The screening of biologically active chemical compound libraries can be an efficient way to reposition Food and Drug Adminstration (FDA)-approved drugs or to discover new therapies for human diseases. Particulate matter with an aerodynamic diameter equal to or less than 2.5 μm (PM_2.5_) is a form of air pollutant that causes significant lung damage when inhaled. This study illustrates drug repositioning with biapenem (BIPM) for the modulation of PM-induced lung injury. Biapenem was used for the treatment of severe infections. Mice were treated with BIPM via tail-vein injection after the intratracheal instillation of PM_2.5_. Alterations in the lung wet/dry weight, total protein/total cell count and lymphocyte count, inflammatory cytokines in the bronchoalveolar lavage fluid (BALF), vascular permeability, and histology were monitored in the PM_2.5_-treated mice. BIPM effectively reduced the pathological lung injury, lung wet/dry weight ratio, and hyperpermeability caused by PM_2.5_. Enhanced myeloperoxidase (MPO) activity by PM_2.5_ in the pulmonary tissue was inhibited by BIPM. Moreover, increased levels of inflammatory cytokines and total protein by PM_2.5_ in the BALF were also decreased by BIPM treatment. In addition, BIPM markedly suppressed PM_2.5_-induced increases in the number of lymphocytes in the BALF. Additionally, the activity of mammalian target of rapamycin (mTOR) was increased by BIPM. Administration of PM_2.5_ increased the expression levels of toll-like receptor 4 (TLR4), MyD88, and the autophagy-related proteins LC3 II and Beclin 1, which were suppressed by BIPM. In conclusion, these findings indicate that BIPM has a critical anti-inflammatory effect due to its ability to regulate both the TLR4-MyD88 and mTOR-autophagy pathways, and may thus be a potential therapeutic agent against diesel PM_2.5_-induced pulmonary injury.

## 1. Introduction

The traditional drug discovery process, with the design and validation of new chemicals, is a time-consuming and expensive process [1,2,3]. Despite the investment in drug discovery, the number of new drugs identified by this classic approach has not increased significantly in the past [1]. Another approach is drug repositioning, which involves identifying new chemicals from old drugs and applying the newly identified drugs to the treatment of a disease other than the drug’s intended disease [4]. An increasing number of companies are scanning existing pharmacopeias and repositioning drug candidates, and several governments are also investing in drug repositioning and related activities [5].

Air pollution from anthropogenic sources has worsened globally, particularly as a result of the development of heavy industry in recent years [6,7]. Suspended particulate matter (PM), less than 2.5 μm (PM_2.5_) in diameter, a well-known indicator of air pollution, has adverse effects on the respiratory and circulatory systems [8]. PM_2.5_ is made up of a number of different components that exert toxic effects, including polycyclic aromatic hydrocarbons, oxygenated volatile organic compounds, and heavy metals [9,10]. The relationship between PM_2.5_ and inflammation has been identified as playing a role in a variety of lung diseases—such as asthma, acute lung injury, and chronic obstructive pulmonary disease—and the secretion of inflammatory cytokines (interleukins (ILs) and tumor necrosis factor (TNF)-α) were induced by PM_2.5_ [11,12,13]. Because there is a significant correlation between exposure to PM_2.5_ and the risk of asthma, and also the incidence and mortality of lung cancer [14], there is a high-priority need to develop new prevention and treatment strategies for respiratory diseases.

**Figure 1:**
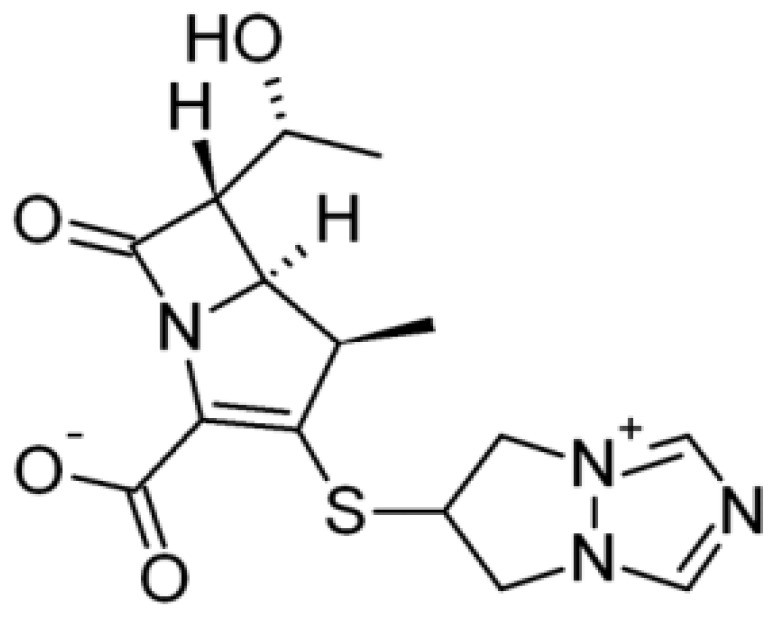
Chemical structure of biapenem (BIPM).

In a study to reposition FDA-approved drugs (1,163 in total), 327 drug candidates associated with pulmonary inflammation were selected. Among the selected chemicals, a high-content screening system (PerkinElmer Operetta, Waltham, Mass.) was used for compound selection. As a result, we found that biapenem (BIPM, Figure 1) had inhibitory effects on PM-induced lung injury. BIPM is a carbapenem antibiotic of antibacterial activity encompassing many Gram-negative and Gram-positive aerobic and anaerobic bacteria, including species producing β-lactamases [15,16]. Previous reports show that BIPM was used for treating pneumonia, by inhibiting bacterial cell wall synthesis and its ability to penetrate into most bacteria [16,17]. However, the effects of BIPM on pulmonary injury, histology, inflammation, and toll-like receptor 4 (TLR4)-autophagy pathways following PM_2.5_ exposure have yet to be investigated. To address this gap in knowledge, a PM_2.5_-exposed mouse model was used to demonstrate our hypothesis that PM_2.5_-induced inflammation and autophagy, and PM_2.5_-induced pulmonary damage, may be controlled by the treatment with BIPM.

## 2. Results

### 2.1. Effects of BIPM on PM_2.5_-Induced Lung Damage

**Figure 2:**
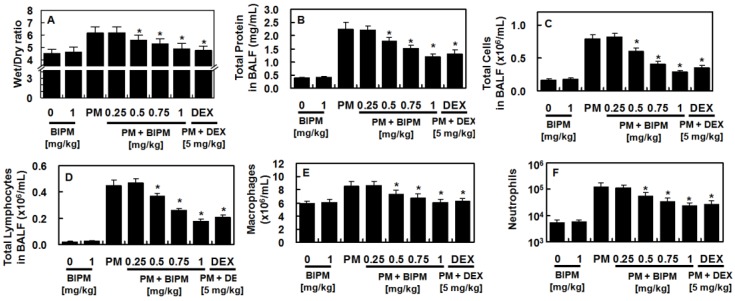
Effects of BIPM on particulate matter (PM)_c_-induced lung damage. The biapenem (BIPM) and dexamethasone (DEX) groups were injected intravenously 30 min after being intratracheally challenged with PM_2.5_ (10 mg/kg in 50 μL of saline). The mice were then sacrificed 24 h post-PM_2.5_-injection and the lung tissue and bronchoalveolar lavage fluid (BALF) were harvested. The effects of various concentrations of BIPM or DEX on (**A**) the wet/dry (W/D) ratio, (**B**) total cells, (**C**) total protein, (**D**) lymphocytes, (**E**) macrophages, and (**F**) neutrophils in the BALF were assessed. The values represent the mean ± SD of three independent experiments. * *p* < 0.01 versus the PM-challenged group.

Measurement of the lung wet/dry (W/D) weight ratio was used to determine the effects of BIPM on PM-induced pulmonary edema. Administration of PM_2.5_ increased the lung W/D weight ratio, which was reduced by BIPM or dexamethasone (DEX) (Figure 2A). Next, we measured the inflammatory cell infiltration and total protein levels in the bronchoalveolar lavage fluid (BALF). Data showed that BIPM or DEX treatment suppressed the PM_2.5_-mediated increase in total protein (Figure 2B), total cell counts (Figure 2C), lymphocyte counts (Figure 2D), macrophage counts (Figure 2E), and the number of neutrophils (Figure 2F), in a dose-dependent manner.

**Figure 3:**
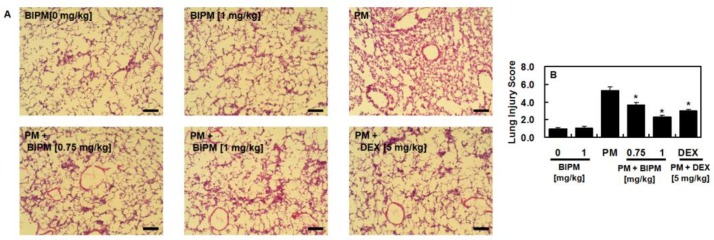
Chemical structure of biapenem (BIPM). Effects of BIPM on PM_2.5_-induced lung histopathological changes. The BIPM and DEX groups were injected intravenously 30 min after being intratracheally challenged with PM_2.5_ (10 mg/kg in 50 μL of saline). The mice were then sacrificed 24 h post-PM_2.5_-injection, and the lung tissue was harvested. (**A**) Lung histology was examined using hematoxylin and eosin staining. Representative images from each group are shown (n = 5). Scale bar: 200 μm. (**B**) Lung injury score. The values represent the mean ± SD of three independent experiments. * *p* < 0.01 versus the PM-challenged group.

To examine the protective effects of BIPM against PM_2.5_-induced lung injury, changes in lung histopathology were investigated using H&E staining. As shown in Figure 3A, enhanced inflammatory cell infiltration and deposition on the alveolar wall by administration of PM_2.5_ were decreased by treatment with BIPM or DEX (Figure 3), indicating that BIPM sufficiently reduced the infiltration of inflammatory cells and protected the lungs from injury by PM_2.5_.

### 2.2. Effects of BIPM on PM_2.5_-Mediated Vascular Barrier Disruption

**Figure 4:**
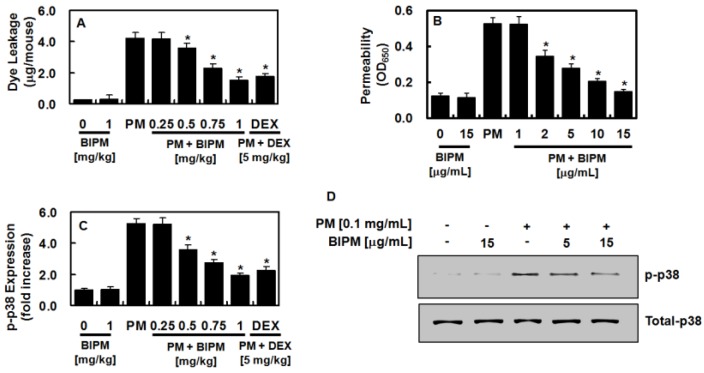
Effects of BIPM on PM_2.5_-induced barrier-disruptive responses and p38 mitogen-activated protein kinase (MAPK) activation. (**A**, **C**, **D**) The BIPM and DEX groups were injected intravenously 30 min after being intratracheally challenged with PM_2.5_ (10 mg/kg in 50 μL of saline). The effects of BIPM or DEX on PM_2.5_-induced vascular permeability were examined by (**A**) measuring the flux of Evans blue in the BALF (expressed as μg/mouse, *n* = 5), (**C**) measuring phospho-p38 expression in purified MLMVECs isolated from each mouse using ELISA, or (**D**) Western blotting. (**B**) The effects of various concentrations of BIPM on PM_2.5_ (0.1 mg/mL, 6 h)-induced barrier disruption were monitored as the flux of Evans blue-bound albumin across MLMVECs. * *p* < 0.01 versus the PM-challenged group.

Because PM has been reported to disrupt the integrity of the vascular barrier [18,19], the effects of BIPM on PM-induced vascular disruptive responses were evaluated. As shown in Figure 4A, dye leakage in the BALF was significantly higher following PM_2.5_ treatment, which was subsequently suppressed by BIPM or DEX. The barrier-protective function of BIPM against PM_2.5_-induced vascular disruptive responses was confirmed in mouse lung microvascular endothelial cells (MLMVECs) (Figure 4B). Because the p38 mitogen-activated protein kinase (MAPK) signaling pathway mediates the vascular damage reaction caused by inflammatory proteins [20,21], we then determined the effects of BIPM on PM_2.5_-induced p38 MAPK activation, finding that PM_2.5_ upregulated the phosphorylation of p38 MAPK, which BIPM treatment significantly inhibited (Figure 4C,D).

### 2.3. Effects of BIPM on PM_2.5_-Induced Pulmonary Inflammation

**Figure 5:**
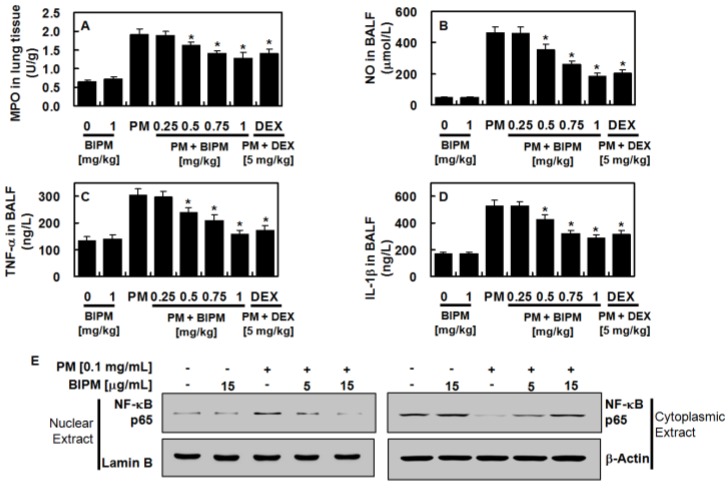
Effects of BIPM on PM-induced pulmonary inflammation. The BIPM and DEX groups were injected intravenously 30 min after being intratracheally challenged with PM_2.5_ (10 mg/kg in 50 μL of saline). The mice were then sacrificed 24 h post-PM-injection, and the lung tissue and BALF were harvested. (**A**) myeloperoxidase (MPO) in lung tissue, (**B**) nitrous oxide (NO), (**C**) TNF-α, and (**D**) IL-1β in the BALF were measured. (**E**) The expression levels of NF-κB in nuclear and cytoplasmic extracts were evaluated with Western blot analyses; actin and lamin B were used as loading controls for the cytoplasmic and nuclear extracts, respectively. The values represent the mean ± SD of three independent experiments. * *p* < 0.01 versus the PM-challenged group.

Because PM_2.5_-induced vascular barrier disruption was inhibited in vivo by BIPM (Figure 4), we next determined the effects of BIPM against PM_2.5_-induced pulmonary inflammatory responses. Inflammatory cytokines such as nitrous oxide (NO), interleukin (IL)-1β (IL-1β), and tumor necsosis factor (TNF)-α (TNF-α) are important indicators of the inflammatory responses, and the degree of neutrophil tissue infiltration is reflected via increased lung myeloperoxidase (MPO) activity. Increased lung tissue MPO activity and NO, IL-1β, and TNF-α production by PM_2.5_ were suppressed by BIPM or DEX treatment (Figure 5 A–D). Additionally, PM_2.5_ increased the expression of p65 nuclear factor (NF)-κb (NF-κB) in the nucleus, but this was inhibited by independent treatment with BIPM in MLMVECs (Figure 5E).

### 2.4. Effects of BIPM on PM_2.5_-Induced Signaling Pathways

**Figure 6:**
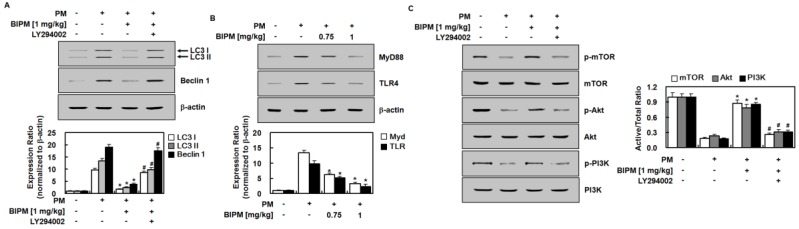
Effects of BIPM on PM-induced signaling pathways. BIPM groups were injected intravenously 30 min after being intratracheally challenged with PM_2.5_ (10 mg/kg in 50 μL of saline). The mice then were sacrificed 24 h post-PM-injection, and the lung tissue was harvested. Representative examples of Western blot analysis demonstrate the expression levels of (**A**) LC3 and Beclin 1; (**B**) TLR4 and MyD88; and (**C**) p-mTOR, mTOR, p-Akt, Akt, p-PI3K and PI3K. Representative images from each group are shown (*n* = 3). The graphs show the densitometric intensities of each protein, normalized to β-actin (**A**,**B**) or total form (**C**). For the blots, *n* = 3. +: treated; −: untreated.

This study investigated the regulatory effects of BIPM on LC3 and Beclin 1 using Western blot analysis. As shown in Figure 6A, LC3 II and Beclin 1 levels were significantly higher in the PM_2.5_-treated group than in the control group. BIPM significantly suppressed the increase in LC3 and Beclin 1 levels induced by PM_2.5_ in mouse lung tissue. This indicates that BIPM can inhibit PM_2.5_-induced autophagy. However, these effects were partially abolished following the administration of LY294002. To understand the mechanisms underlying the anti-PM_2.5_-induced inflammatory and anti-autophagy effects of BIPM, both the TLR4 and the mammalian target of rapamycin (mTOR)-autophagy pathways were investigated using Western blotting for TLR4, MyD88, p-mTOR, total mTOR, p-Akt, Akt, p-PI3K, and phosphoinositide 3-kinase (PI3K) in mouse lung tissue. The intratracheal instillation of PM_2.5_ upregulated the expression of TLR4 and MyD88 in this tissue (Figure 6B), which was subsequently reduced by treatment with BIPM (1 mg/kg). Compared with the control group, the levels of p-mTOR, p-Akt, and p-PI3K were significantly lower in the PM_2.5_ group (Figure 6C). In addition, BIPM treatment significantly restored the levels of p-mTOR, p-Akt, and p-PI3K, demonstrating that BIPM can activate the PI3K/Akt/mTOR pathway. However, LY294002 significantly reversed these effects. Additionally, no significant differences in the total levels of mTOR, Akt, or PI3K were observed between the four groups.

## 3. Discussion

In the current study, we were interested in the potential application of BIPM in the treatment of PM_2.5_-induced lung injury. Previous experimental research has shown that PM increases the inflammatory response of endothelial cells, epithelial cells, and macrophages, leading to local lung inflammation [22,23,24]. Additionally, overexpression of inflammatory inducers could cause systemic inflammatory responses and deteriorate other organs [25]. Therefore, inflammatory responses are recognized as the dominant biological response to PM exposure. Recently, we reported PM_2.5_-mediated pulmonary inflammatory responses such as the vascular disruptive responses and the upregulated expressions of inflammatory molecules such as p38, reactive oxygen species (ROS), IL-6, and TNF-α [26,27,28]. The present research demonstrated that BIPM can inhibit both the infiltration of lung tissue by inflammatory cells and inflammatory cytokine production in our mouse model of PM-induced lung injury. The possible mechanisms underlying the anti-PM_2.5_-induced inflammatory effect of BIPM are the reduction of TLR4 and MyD88 expression, the increase in mTOR phosphorylation, and the prevention of autophagy. Our study employed dexamethasone as a positive control because it is the most frequently used anti-inflammatory agent in the treatment of lung injury [29,30,31].

Autophagy is a lysosome-dependent process that collects damaged organelles, protein aggregates, and degraded cytoplasmic material in autophagic vacuoles [32]. Autophagy has been shown to be involved in the developmental and regulatory processes of lung injury [33]. In intact lung tissue, mTOR is known to be activated while autophagy-related protein LC3 II is downregulated [34], while following lung injury, the suppression of mTOR is accompanied by the upregulation of LC3 II in human bronchial epithelial cells [35]. In addition, when TLR4 or MyD88 is knocked down, lipopolysaccharide (LPS)-induced mTOR phosphorylation is downregulated, indicating that mTOR activation is caused by the TLR4-Myd signaling pathway and that LPS could inhibit the autophagy [34]. Although autophagy may be involved in anti-inflammatory responses, autophagy may not play a significant role in LPS-induced inflammation since rafamycin treatment could improve pulmonary injury after LPS infection by down-regulating mTOR. As a result, there is a strong possibility of a signaling network between TLR4 and autophagy in the presence of PM-induced lung injury, with autophagy governed by a complex signaling network, and TLR4, a critical sensor of autophagy that is significantly involved in PM-induced immunity responses [36,37]. Since mTOR functions as a key autophagy checkpoint and is involved in PM-induced pulmonary inflammatory responses, it has been suggested that both the mTOR/autophagy and TLR4/MyD88 pathways affect lung injury [35]. The TLR4-MyD88 pathway is considered an upstream signaling mediator of PM-induced pulmonary inflammatory responses, which induces the secretion or production of inflammatory cytokines and oxidants [36]. Oxidizing agents or other cytokines can inhibit mTOR activation, cause tissue cell autophagy, and lead to excessive inflammation and tissue damage [38]. Autophagy can also be regulated by multiple signaling pathways including the PI3K/Akt pathway [39], which is a key regulator of cell growth and survival that helps to mediate cardiomyocyte survival [40].

As a pivotal autophagy regulator, mTOR is phosphorylated by the activation of the PI3K/Akt pathway [39]. Once mTOR is activated, it can protect pulmonary tissues against, or promote their recovery from, lung injury by reducing autophagy [41,42]. Regarding this, our results showing that BIPM increased levels of p-mTOR/p-PI3K/p-Akt and decreased levels of LC3 II/Beclin 1 indicate that BIPM inhibits excessive autophagy through the activation of the PI3K/Akt/mTOR pathway. Furthermore, our Western blot experiments showed that BIPM reduced TLR4 and MyD88 expression (Figure 6B), indicating that BIPM inhibits PM-induced TLR4 and MyD88 upregulation, thus reducing inflammatory cytokines (e.g., IL-1β and TNF-α) and the production of oxidants (e.g., MPO and NO), which in turn activate mTOR and the autophagy of tissue cells.

It is important to note that PM is generated directly from a variety of sources, such as construction sites, smokestacks, fires, and unpaved roads. PM particles have many sizes and morphologies, and PM pollution can be a combination of hundreds of different compounds. Therefore, a limitation of this study is that it does not address whether BIPM inhibits the pulmonary damage caused by different PM compounds.

In the present study, our results indicate that BIPM attenuated PM_2.5_-induced pulmonary damage, including reducing the lung W/D weight ratio, total protein levels, the numbers of lymphocytes, inflammatory cell infiltration, inflammatory cytokine expression, and hyperpermeability. Moreover, BIPM enhanced the recovery of tissue from damage caused by PM_2.5_-induced lung injury by inhibiting the TLR4 and autophagy pathways. The evaluation of BIPM’s effects on PM_2.5_-induced inflammation and the TLR4 and autophagy pathways will enlighten us as to the application of BIPM in addressing diesel PM_2.5_-medicated adverse health effects. Therefore, this study could contribute to the development of new prevention and treatment strategies for PM-induced respiratory diseases, indicating that BIPM can be used as a potentially efficient therapeutic agent against PM_2.5_-induced lung injury.

## 4. Materials and Methods

### 4.1. Reagents

BIPM was obtained from Santa Cruz Biotechnology Inc. (Santa Cruz, CA, USA). Diesel PM NIST 1650b [43] was obtained from Sigma-Aldrich Inc. (St. Louis, MO, USA), and was mixed with saline and sonicated for 24 h to avoid the agglomeration of suspended PM_2.5_ particles. As a positive control, dexamethasone (DEX) was used (Sigma-Aldrich Inc, St. Louis, MO, USA).

### 4.2. Animals and Husbandry

Seven-week-old male Balb/c mice (approximate body weight of 27 g), purchased from Orient Bio Co. (Sungnam, Republic of Korea), were used after 12 days of acclimatization. The mice were treated in accordance with the Guidelines for the Care and Use of Laboratory Animals of Kyungpook National University (IRB #: KNU2017-102, January, 2017). Eighty mice were randomly divided into eight groups, each consisting of 10: a mock control group, a BIPM control group, a PM_2.5_ group, PM+ BIPM (0.25, 0.5, 0.75, and 1 mg/kg) groups, and a DEX group (5 mg/kg). The mice in the control group received an equal volume of phosphate buffered saline (PBS). Mice in the BIPM or DEX groups were injected intravenously after the intratracheal instillation of PM_2.5_ (10 mg/kg mouse body weight in 50 μL of saline). One day after injection, the mice were sacrificed and bronchoalveolar lavage fluid (BALF) and lung tissue were collected for further study. The intratracheal instillation of PM_2.5_ has been previously shown to cause lung damage, including increased vascular permeability, alveolar epithelial dysfunction, and vascular inflammation [14,44]. Thus, administration of PM via intratracheal instillation is a convenient and effective way to induce lung injury in vivo.

### 4.3. Primary Culture of Mouse Lung Microvascular Endothelial Cells (MLMVECs)

MLMVECs were acquired using a modified version of a previous approach [28].

### 4.4. Lung Wet/Dry Weight Ratio

The right lung was weighed to obtain wet weight. Then, the lungs were dried in an oven at 120 °C for one day and weighed again to obtain dry weight. Pulmonary edema was determined by calculating the wet/dry weight of the lung (W/D).

### 4.5. Hematoxylin and Eosin (H&E) Staining

After lung was removed, washed, and fixed with a 4% formaldehyde solution (Junsei, Tokyo, Japan), samples were dehydrated, embedded in paraffin, sliced (4-μm thick), deparaffinized, rehydrated, and stained with hematoxylin as described previously [28,45].

### 4.6. ELISA of Phosphorylated p38 MAPK, MPO, NO, IL-1 β, and TNF-α

The levels of phosphorylated p38 mitogen-activated protein kinase (MAPK) in the MLMVEC lysates were analyzed using a commercially available enzyme-linked immunosorbent assay (ELISA) kit (Cell Signaling Technology, Danvers, MA, USA). The concentrations of myeloperoxidase (MPO), nitrous oxide (NO), interleukin (IL)-1β and tumor necrosis factor (TNF)-α in the BALF were determined using manufacturer-suggested ELISA kits (R&D Systems, Minneapolis, MN, USA) by using an ELISA plate reader (Tecan Austria GmbH, Grödig, Austria).

### 4.7. Protein Concentration and Cell Count in the BALF

After centrifugation at 3000 rpm for 10 min at 4 °C, the BALF supernatant was used to assess the total protein concentration with a QuantiPro™ BCA Assay Kit (Sigma-Aldrich Inc.), and the cytokine levels were measured. Resuspended cells in PBS were measured using a hematology analyzer.

### 4.8. Permeability Assays

For spectrophotometric quantification of MLMVEC permeability in response to increasing concentrations of PIPM in vitro, the flux of Evans blue-bound albumin across functional cell monolayers was measured using a modified two-compartment chamber model, as previously described [46,47,48]. For in vivo assays, mice in the BIPM or DEX groups were injected intravenously after the intratracheal instillation of PM_2.5_ (10 mg/kg mouse body weight in 50 μL of saline) as described above. For anesthesia, a 2% isoflurane-oxygen mixture (Forane; JW Pharmaceutical, Seoul, Korea) was delivered using a gas anesthesia machine (RC2 Rodent Circuit Controller; VetEquip, Pleasanton, CA, USA). The mice were first anesthetized in a respiratory chamber, then anesthetized through a face mask, followed by an intravenous injection of a 1% solution of Evans blue dye in saline. Six hours later, mice were euthanized by cervical dislocation and BALF was collected. In vivo permeability was measured using an ELISA plate reader as described previously [46,47,48].

### 4.9. Western Blot Analysis

Regular Western blotting analysis was conducted as described previously [28] using the following primary antibodies: anti-light chain (LC)3 (1:1000), Beclin 1 (1:1000), TLR4 (1:1000), MyD88 (1:1000), mTOR (1:1000), phosphorylated (p)-mTOR (1:1000), p38 (1:1000), (p)-p38 (1:800), NF-kB p65 (1:1000), Akt (1:1000), p-Akt (1:2000), p-PI3K (1:1000), and PI3K (1:800) (Cell Signaling Technology, Inc., Danvers, MA, USA). Densitometry analysis was performed using the ImageJ Gel Analysis tool (NIH, Bethesda, MD, USA).

### 4.10. Statistical Analysis

Data are presented as the mean ± standard deviation (SD), using SPSS for Windows version 16.0 (SPSS, Chicago, IL, USA). Differences among groups were evaluated using a one-way analysis of variance (ANOVA) followed by Dunnett’s tests. Values of *p* < 0.05 were considered statistically significant.
